# Salivary MMP-9 in the detection of oral squamous cell carcinoma

**DOI:** 10.4317/medoral.21626

**Published:** 2017-02-04

**Authors:** Andre Peisker, Gregor F. Raschke, Mina D. Fahmy, Arndt Guentsch, Korosh Roshanghias, Joschka Hennings, Stefan Schultze-Mosgau

**Affiliations:** 1 MD, DMD. Department of Cranio-Maxillofacial & Plastic Surgery, Jena University Hospital, Jena, Germany; 2 MD, DMD, PhD. Department of Cranio-Maxillofacial & Plastic Surgery, Jena University Hospital, Jena, Germany; 3 BSc. Department of Surgical Sciences, Marquette University, School of Dentistry, Milwaukee, Wisconsin, USA; 4 DMD, PhD, MHBA. Department of Surgical Sciences, Marquette University, School of Dentistry, Milwaukee, Wisconsin, USA; 5 DMD. Department of Cranio-Maxillofacial & Plastic Surgery, Jena University Hospital, Jena, Germany; 6 Department of Cranio-Maxillofacial & Plastic Surgery, Jena University Hospital, Jena, Germany; 7 MD, DMD, PhD. Department of Cranio-Maxillofacial & Plastic Surgery, Jena University Hospital, Jena, Germany

## Abstract

**Background:**

Oral squamous cell carcinoma (OSCC) is the most common malignant tumour of the oral cavity. Detection of OSCC is currently based on clinical oral examination combined with histopathological evaluation of a biopsy sample. Direct contact between saliva and the oral cancer makes measurement of salivary metalloproteinase- 9 (MMP-9) an attractive alternative.

**Material and Methods:**

In total, 30 OSCC patients and 30 healthy controls were included in this prospective study. Saliva samples from both groups were collected, centrifuged and supernatant fluid was subjected to ELISA for assessment of MMP-9. The median salivary MMP-9 values with interquartile range (IQR) of OSCC patients and the control group were statistically analysed using the Mann-Whitney U-test. The receiver operating characteristic (ROC) curve was constructed and the area under curve (AUC) was computed.

**Results:**

The median absorbance MMP-9 value of the OSCC group was 0.186 (IQR=0.158) and that of control group was 0.156 (IQR=0.102). MMP-9 was significantly increased in the OSCC patients than in the controls by +19.2% (*p*=0.008). Median values in patients with recurrence and in patients with primary event were 0.233 (IQR=0.299) and 0.186 (IQR=0.134) respectively. MMP-9 was significantly increased in patients with primary event (*p*=0.017) compared to controls by +19.2%. No significant increase of MMP-9 level was detected when comparing patients with recurrence and healthy controls (+49.4%; *p*=0.074). The sensitivity value of MMP-9 was 100% whereas the specificity value was 26.7% with AUC of 0.698.

**Conclusions:**

The present data indicates that the elevation of salivary levels of MMP-9 may be a useful adjunctive diagnostic tool for detection of OSCC. However, further studies are necessary to provide scientific and clinical validation.

** Key words:**Oral squamous cell carcinoma, oral cancer, saliva, salivary diagnostics, cancer detection, MMP-9, metalloproteinases.

## Introduction

More than 95% of oral cancers are squamous cell carcinomas (OSCC) (1). Despite great progress in cancer treatment throughout the last several decades, the prognosis of OSCC is still poor. Although the oral cavity is easily accessible for direct visual examination, most OSCC patients are in an advanced stage, which is believed to be a major reason for the low survival rate of about 50 to 63% (2-4). In contrast, early oral cancers are often highly curable with less morbidity than cancers that are in later stages (5). These statistics emphasise the importance of early and accurate detection by clinicians.

Currently, the detection and diagnosis of OSCC is based on clinical oral examination combined with histopathological evaluation of a biopsy sample. In response to the need for early detection of oral cancer, several diagnostic adjuncts have been developed, including the use of salivary biomarkers (6-8). Collecting saliva is a relatively simple procedure and is non-invasive, compared to biopsying or drawing blood.

To date, more than 100 potential OSCC salivary biomarkers have been suggested in the current literature (9). One of these potential biomarkers is metalloproteinase-9 (MMP-9). MMP-9 is a class of enzymes that has been shown to be associated in cancer pathogenesis as they are involved in the degradation of extracellular matrix. Significant differences have been found in MMP-9 levels between the OSCC patients and their respective control groups (10,11).

The purpose of this study was to determine the levels of MMP-9 in saliva of patients with OSCC and those in a healthy control group, and to investigate the diagnostic value of MMP-9.

## Material and Methods

This prospective study was initiated after the local ethics committee of the Jena University Hospital gave its approval (4369-03/15). Informed written consent was obtained from all individual participants included in the study.

- Collection of samples

A tissue biopsy sample for histological examination was obtained from patients with potentially malignant disease after the salvia collection. If a biopsy sample was negative in the experimental group, the patient was

excluded from the study.

A total of 60 samples were included in the dataset, including 30 cases of OSCC and 30 of healthy individuals. The controls were gender and age-matched subjects enrolled during the same period when OSCC subjects were recruited. Controls were generally healthy individuals free from any systemic disease, cancer or inflammatory oral lesions.

- Study population

The demographic data of all subjects included in this study and the tumour clinicopathological parameters of patients with OSCC are listed in  Table 1. The data obtained included age, gender, tumour site, tumour stage according to the TNM classification of the International Union against Cancer (UICC) and histological tumour grade.

**Table 1:**
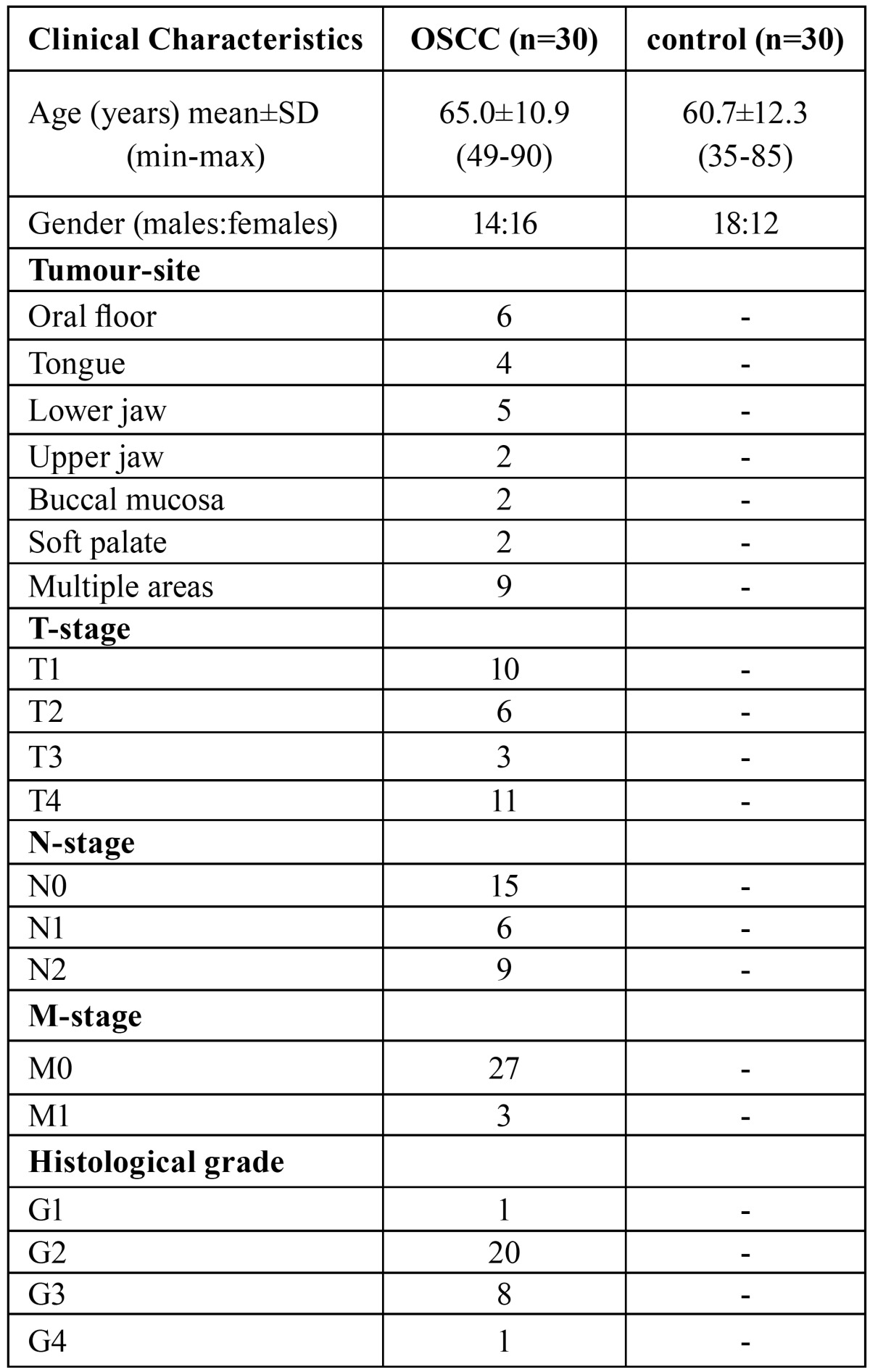
Demographic and Clinical Data of Subjects.

Among the 30 OSCC patients of the study group, 16 were females and 14 were males. The mean age was 65.0±10.9 years (mean±SD) with a range from 49 to 90.

The control group included 12 females and 18 males, mean age 60.7±12.3 years (range 35-85).

- Saliva collection and immunoreactivity assay for salivary

In both groups, saliva was collected in the morning between 7 and 8 o’clock before food intake. Stimulated saliva samples were collected using Salivette® (Sarstedt, Nuernbrecht, Germany). Patients were asked to make chewing motions for 30 seconds to stimulate saliva production.

MMP-9 concentrations were measured by immunoreactivity assay similar to the method previously described (10,11). In brief, following the collection, the saliva was immediately centrifuged at 1,000 g at 20°C for 2 min and the resulting supernatant was used to determine the total amount of total protein with BCA Protein Assay Kit (Thermo Scientific, Rockford, IL, USA) as specified by the manufacturer.

After calculation of the concentration of total protein, a solution with 10 ng total protein pro μl of each sample was made by diluting the saliva with PBS (Invitrogen, Camarillo, CA, USA). Thereafter, 100 μl of each sample was added to Nunc Immuno Plate wells (Thermo Fisher Scientific Nunc A/S, Roskilde, Denmark). The plate was covered and stored overnight at 4°C.

The next day, each well was washed 3 times with 100 μl PBS-T (PBS containing 0.05% Tween 20) and a volume of 100 μl blocking solution (PBS-T containing 1% BSA) was added to each well. After 2 hour incubation at room temperature, the blocking solution was removed and 100 μl of primary antibody (polyclonal rabbit anti human MMP-9, biorbyt, San Francisco, CA, USA) diluted 1:20,000 in blocking solution was added to each well. After another 2 hour incubation at room temperature, the plate was washed as described before and a volume of 100 μl of secondary antibody (peroxidase-conjugated goat anti-rabbit IgG, Jackson Immuno Research, West Grove, PA, USA) diluted 1:5,000 in blocking solution was added to each well. Finally, after a third 2 hour incubation period at room temperature the plate was washed as described before. To achieve colour development, 100 μl of 3.3`, 5.5`-tetramethylbenzidine solution (Southern Biotech, Birmingham, AL, USA) was added to each well. After 2 min, 100 μl of TMB Stop Solution (Southern Biotech, AL, Birmingham, USA) was added to each well. Absorbencies of the samples, representing the levels of MMP-9, were measured at a wavelength of 450 nm directly after the addition of the Stop Solution, using a ThermoMax microplate reader (Molecular Devices, Sunnyvale, CA, USA).

- Statistical analysis

The nonparametric Mann-Whitney U test was used to compare the differences in the median absorbance values in the groups. Receiver operating characteristic (ROC) curves were used to evaluate the diagnostic effectiveness of the potential biomarker and to find an optimal cut-off point based on the maximum corresponding sensitivity and specificity. Furthermore, the area under curve (AUC) presented a direct measure of the diagnostic accuracy of the test. Levels of statistical significance have been calculated at the 5% level of probability (*p* <0.05). Statistical analyses were performed using SPSS 21.0 for Windows (Chicago, IL).

## Results

**Figure 1:**
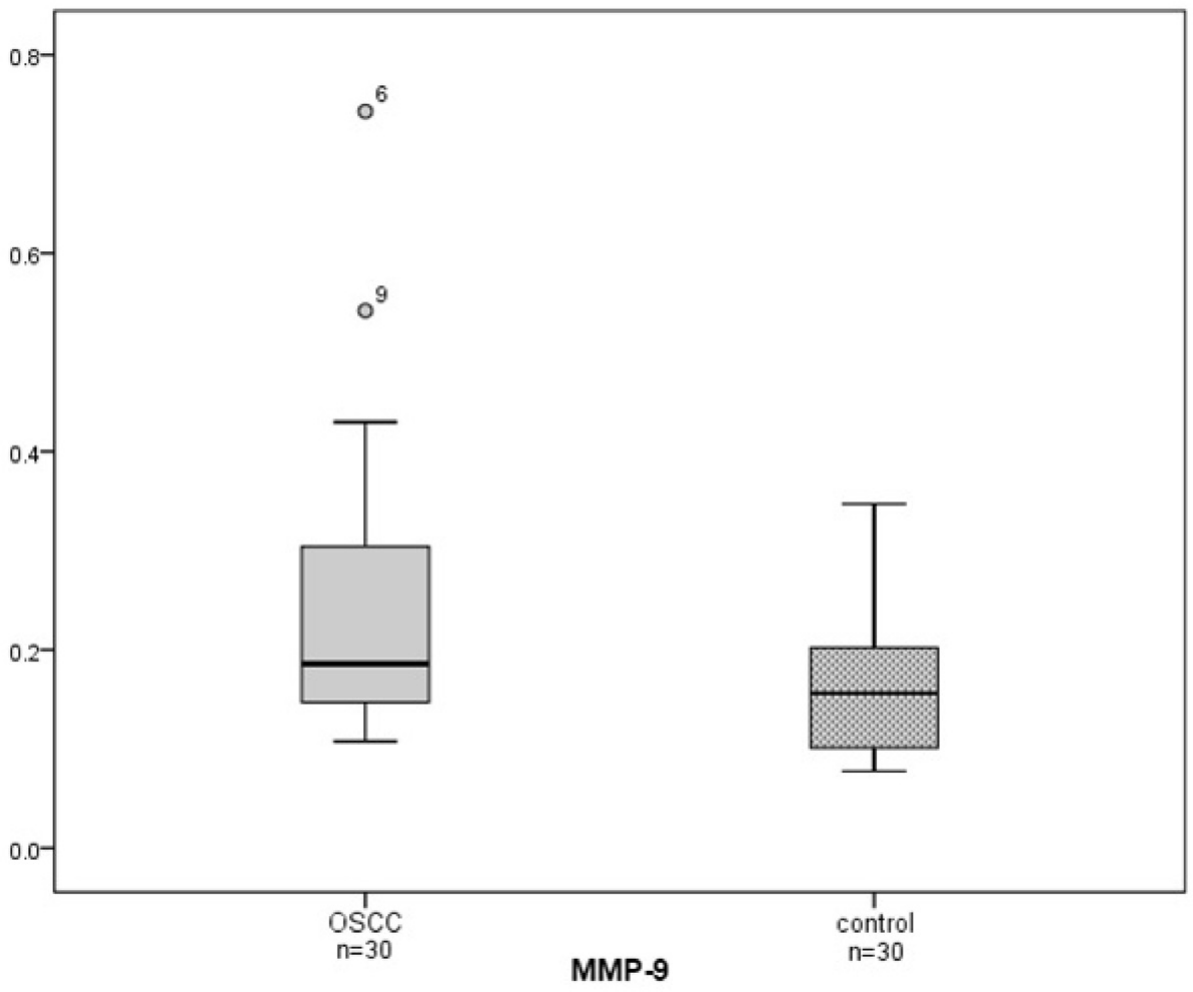
Box plots of median absorbance MMP-9 values from the OSCC patients and the controls. Salivary MMP-9 was significantly increased in the OSCC patients compared to the control group (*p*=0.008).

The saliva analysis showed differences in the level of MMP-9. The median absorbance value in controls was 0.156 with interquartile range (IQR) of 0.102. In contrast, the median absorbance value in the study group was 0.186 (IQR=0.158). Thus, MMP-9 was significantly increased in the OSCC patients by +19.2% (*p*=0.008).

(Fig. 1) shows box plots of the two groups. Furthermore, median absorbance values in patients with recurrence and in patients with primary event were 0.233 (IQR=0.299) and 0.186 (IQR=0.134), respectively. In patients with primary event, MMP-9 was significantly higher by +19.2% (*p*=0.017) compared to controls. In contrast, no significant differences of MMP-9 levels were seen when comparing patients with recurrence and healthy controls (+49.4%; *p*=0.074).

**Figure 2:**
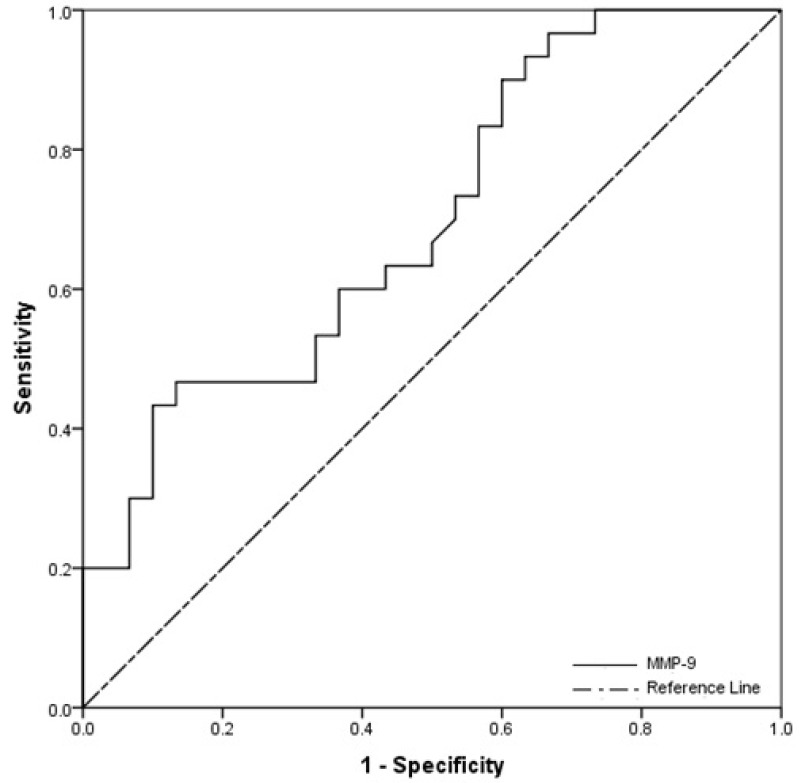
ROC analysis of salivary MMP-9 in diagnosis of OSCC for the OSCC patients vs. healthy controls.

As shown in fig. 2, the ROC curve was created to demonstrate the predictive power of MMP-9 for OSCC. The AUC was 0.698 (cut off value > 0.104; sensitivity 100%; specificity 26.7%; 95% confidence interval, 0.567-0.830; *p*=0.008) for OSCC patients vs. healthy controls.

## Discussion

Most OSCCs are found when the cancer has developed into the advanced stages, which is believed to be a major reason for low survival rates (2-4). In contrast, early detection and diagnosis leads to a greater survival rate and plays an important role in successful clinical treatment (5). Early diagnosis of OSCC is difficult because the early stages may be painless and often a burning sensation may not develop until the malignant lesion has appreciably advanced (5,12). Detection and diagnosis of OSCC is currently based on clinical oral examination combined with biopsy for a histopathological examination if a suspect area is detected (1). The location from which the tissue biopsy sample is taken is essential for histological verification of the oral cancer. However, selecting the correct site for biopsy is difficult due to the non-uniform appearance of premalignant and malignant lesions (5,12).

Today, the presence of OSCC salivary biomarkers is well known based on the direct contact between saliva and the premalignant or malignant lesions. Saliva sampling and processing is a simple procedure and is a more easily accessible fluid than tissue biopsies and blood sampling. Furthermore, saliva sampling eliminates risks of contamination and infection (10). The screening and detection of early OSCC lesions using saliva seems to be promising. Currently, more than 100 potential salivary biomarkers have been reported in the literature (9). In the present study, we analyzed the levels of MMP-9 in saliva of patients with OSCC and in saliva of patients in the healthy control group. MMP-9 is a class of zincdependent proteinases (13) that have been shown to associate in numerous pathological processes as they degrade type IV collagen, a major component of the basal lamina, and other types of collagens (V, VII and X), elastin and fibronectin. Studies have reported a high expression in stromal cells surrounding the invading front of metastasizing tumours. In addition, an elevation of their levels in tumour endothelium and in urine of cancer patients has been found (14-16). Furthermore, MMP-9 may play a significant role in angiogenesis (17-

19). Thomas *et al.* showed that MMP-9 expression *in vitro* is modulated by integrin alpha v beta 6, which is expressed only in OSCC and not in the normal oral epithelium (20). Kosunen *et al.* reported that strong stromal MMP-9 staining intensity was correlated with poor tumour differentiation (21).

In our study, MMP-9 level in the OSCC patients compared to healthy controls was altered in a highly significant manner (+19.2%). Furthermore, it was characterised by high sensitivity (100%) and moderate specificity (26.7%) values. The current data showed a cut off value of > 0.104. This means that cases above these cut off values can be diagnosed as having OSCC, whereas cases below these values can be diagnosed as negative (healthy) cases.

Shpitzer *et al.* corroborated these results, as they also note that MMP-9 increased in OSCC patients compared to controls by 35% to 39% (10,11). Thus, MMP-9 could be used as a diagnostic adjunct for early detection of oral cancer.

Patients with OSCC have a high risk of tumour relapse.

In the current literature, a recurrence rate from 29.8% to 47.1% has been reported (22-24) with local recurrence being more common (22). Therefore, an improved diagnostic tool to predict which patient is most at risk for tumour relapse is needed (25). In addition to clinical examinations and routine imaging modalities such as ultrasonography, computed tomography or magnetic resonance imaging (22), salivary MMP-9 could be of special importance for patient monitoring during follow-up. In the present study, median absorbance MMP-9 value in patients with recurrence was highly increased by 49.4% compared to healthy controls, but there was no significant difference (*p*=0.074). Thus, measuring salivary MMP-9 levels cannot be recommended in the routine diagnostic assessment for tumour recurrence during follow-up.

## Conclusions

Salivary diagnostics for OSCC are very promising due to the direct contact of saliva with oral cancer lesions. MMP-9 seems to play an important role in OSCC pathogenesis. The results of the present study suggested that cancer-related changes in salivary MMP-9 could be used as a tool for early detection of OSCC. Further studies in large patient cohorts are necessary before salivary MMP-9 might be used in future clinical diagnostic applications.
